# Regulation and imaging of gene expression *via* an RNA interference antagonistic biomimetic probe†
†Electronic supplementary information (ESI) available: RISC assay in cell extract, qRT-PCR assay, western blot assay, feasibility and universality of the strategy assay, and specific measurement of RISC. See DOI: 10.1039/c7sc00909g



**DOI:** 10.1039/c7sc00909g

**Published:** 2017-05-05

**Authors:** Kai Zhang, Xue-Jiao Yang, Wei Zhao, Ming-Chen Xu, Jing-Juan Xu, Hong-Yuan Chen

**Affiliations:** a State Key Laboratory of Analytical Chemistry for Life Science and Collaborative Innovation Center of Chemistry for Life Sciences , School of Chemistry and Chemical Engineering , Nanjing University , Nanjing 210023 , China . Email: weizhao@nju.edu.cn ; Email: xujj@nju.edu.cn; b Key Laboratory of Nuclear Medicine , Ministry of Health , Jiangsu Key Laboratory of Molecular Nuclear Medicine , Jiangsu Institute of Nuclear Medicine , Wuxi , Jiangsu 214063 , China

## Abstract

Regulation of gene expression is highly important in the area of cell biology. In this work a novel convenient and versatile strategy is reported which permits both gene regulation and imaging in living cells. An oligonucleotide-based biomimetic probe was designed to target an RNA-induced silencing complex (RISC) and served as an agent for the modulation of c-Myc protein expression in living cells through regulating the RNA interference (RNAi) pathway. In this probe, a DNA strand (Strand1) serving as the frame was immobilized on a AuNP with a thiol group at the 5′ end. Strand2, designed to recognize the target RISC with an RNA fragment, was hybridized with the complementary sequence of Strand1. In the original state, the fluorescence of the Cy3 modifier at the 5′ end of Strand2 was quenched by both the AuNP and BHQ2, which labelled the 3′ end of Strand1. In the presence of RISC, Strand2 was cleaved, resulting in a shorter oligo part with a corresponding lower melting temperature than that of the original full-length Strand2. The shorter oligonucleotide strand containing the Cy3 fluorophore was released, accompanied by a recovered fluorescence signal. Through evaluating the fluorescence intensity, the competition for RISC was dynamically monitored in single cells. Furthermore, capturing RISC by this probe resulted not only in restored fluorescence intensity but also increased c-Myc oncogene expression. Hence, gene expression could be selectively and precisely regulated and imaged *via* the RISC targeting probe. The synthetic method for the biomimetic probe is universally applicable, and facilitates the fundamental study of RNAi pathways, or development of a gene regulation strategy without cytokine activation. The gene regulation and imaging strategy will accelerate the unveiling of the basic role of the RISC cleavage interaction, the mystery of RNA-silencing and therapeutic monitoring of cancer.

## Abstract 2

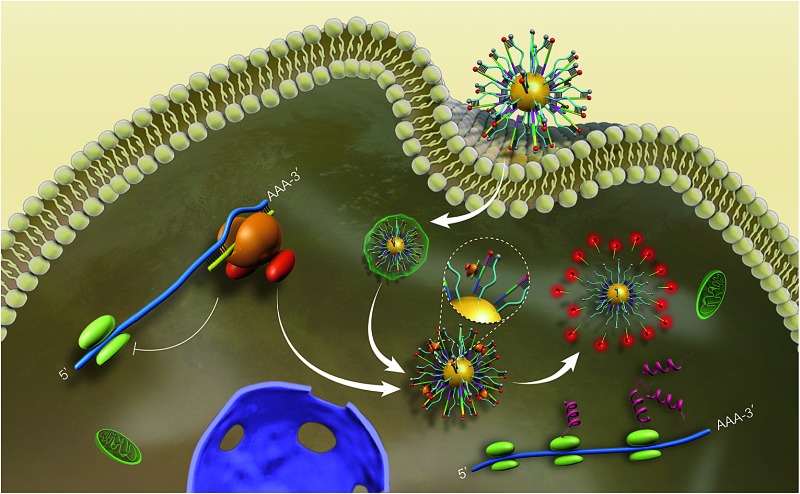
A versatile strategy is reported which permits gene regulation and imaging in living cells *via* an RNA interference antagonistic probe.

## Introduction

Gene expression can be regulated by various cellular pathways with a wide range of mechanisms, the study of which has a significant impact on research into gene pathways and functions.^1–3^ The RNA-induced silencing complex (RISC), a generic term for a family of heterogeneous molecular complexes that can be programmed to target specific genes, is a powerful regulator of gene expression.^4^ RISC contains Argonaute (Ago) protein and small-interfering RNA (siRNA), a small regulatory RNA in the cytoplasm of a eukaryotic cell.^5^ The siRNA assembled into RISC guides the complex to complementary mRNA through base-pairing interactions.^6^ Then the other core component of RISC, Ago protein, which offers a binding site for siRNA in the RISC assembly, functions as a slicer to cleave target mRNA and results in RNA degradation. Studying the RISC mediated gene-regulation facilitates the investigation of both RNA interference (RNAi) pathways and siRNA based therapies.

In order to investigate the dynamic process of gene regulation in living cells, *in situ* determination of gene expression levels is required. However, the most commonly used techniques of quantitative reverse transcriptase polymerase chain reaction (qRT-PCR) and western blot are carried out with cell extracts.^7,8^ A pioneering approach uses green fluorescent protein (GFP),^9–11^ the fluorescence of which changes upon different levels of the *gfp* (mut2) gene being expressed in the host of the target gene plasmid, for *in situ* imaging. Since the chromophore of GFP is produced through an intracellular posttranslational autocatalytic cyclization that does not require any cofactors or substrates, fusion of GFP to a protein rarely affects the gene expression pathway.^12^ However, the extensive application of GFP has been hampered by the complex design of specific plasmids for specific target genes.^12^ Thus, development of a versatile and robust technique for visualizing endogenous gene regulation in living cells is of great interest.

**Scheme 1:**
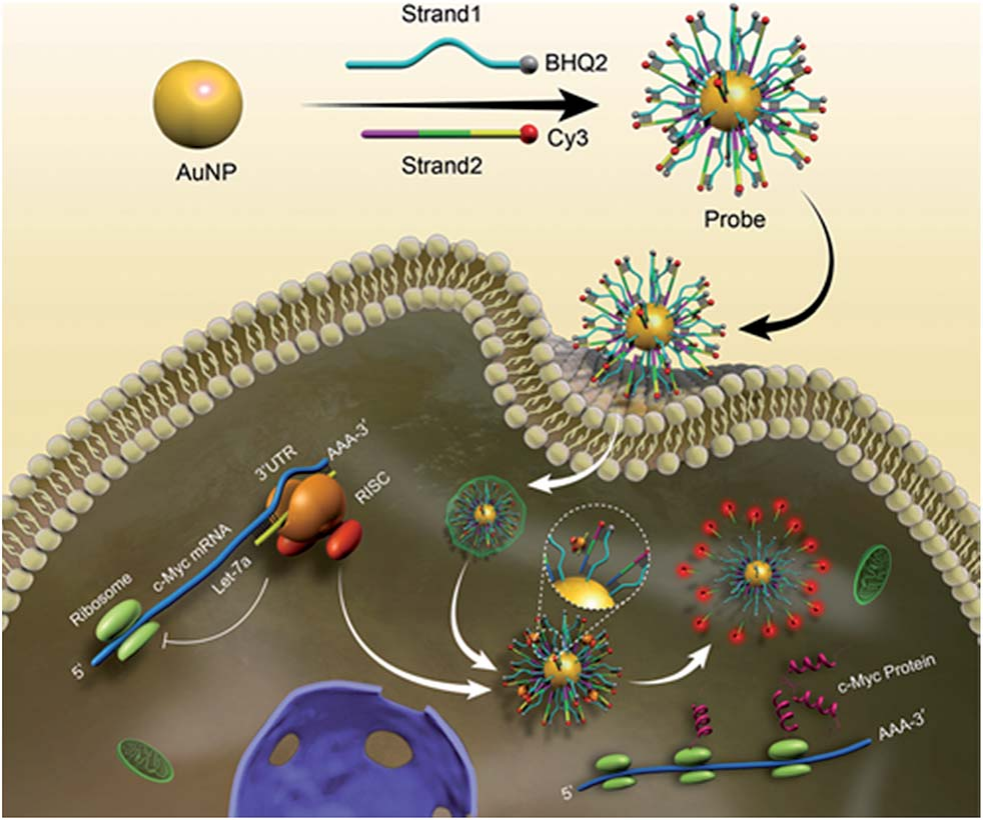
Schematic representation of the biomimetic probe regulating and imaging gene expression by interference in the RNA silencing process. After incubation of antagonistic biomimetic probes, the competitive access to RISC resulted in an increased c-Myc expression and restored fluorescence intensity.

Herein, we propose a strategy for both regulating the c-Myc oncogene *via* antagonistic RISC, and monitoring the dynamic process *in situ*. C-Myc oncogene, a regulator gene that codes for a transcription factor, serves as a potent inducer of both cell proliferation and apoptosis.^13^ It was discovered that Let-7a regulated the expression of the c-Myc oncogene through the antisense oligonucleotide regulation model.^14,15^ In recent years, biomimetic probes with multiple cellular functions have been adopted as intracellular machinery with high efficiency and limited side effects.^8^ In this work, to achieve the goal of gene regulation and imaging at the same time, we synthesized a fluorophore labelled biomimetic probe using a gold nanoparticle (AuNP) chemically functionalized with alkylthiol-terminated oligonucleotides for cellular delivery, which specifically targeted Argonaute2 (Ago2)/Let-7a (antisense oligonucleotide of c-Myc mRNA) and released fluorophores in living cells (Scheme 1). In our work, through evaluating the fluorescence intensity, the competition for RISC was dynamically monitored in single cells. We found that capturing RISC resulted in increased c-Myc expression and restored fluorescence intensity. Also, gene expression could be selectively and precisely regulated and imaged at multi-levels *via* the RISC targeting probe. In contrast to conventional antisense oligonucleotide-based therapies relying on cellular machinery,^16–19^ regulating gene expression *via* a biomimetic RISC targeting probe could limit potential side effects. The *in situ* study reveals that Let-7a regulates c-Myc gene expression *via* the RNA interference (RNAi) pathway. The well-designed biomimetic probe for gene regulation and imaging will accelerate the unveiling of the basic role of the RISC cleavage interaction, the mystery of RNA-silencing and therapeutic monitoring of cancer.

## Results and discussion

**Fig. 1:**
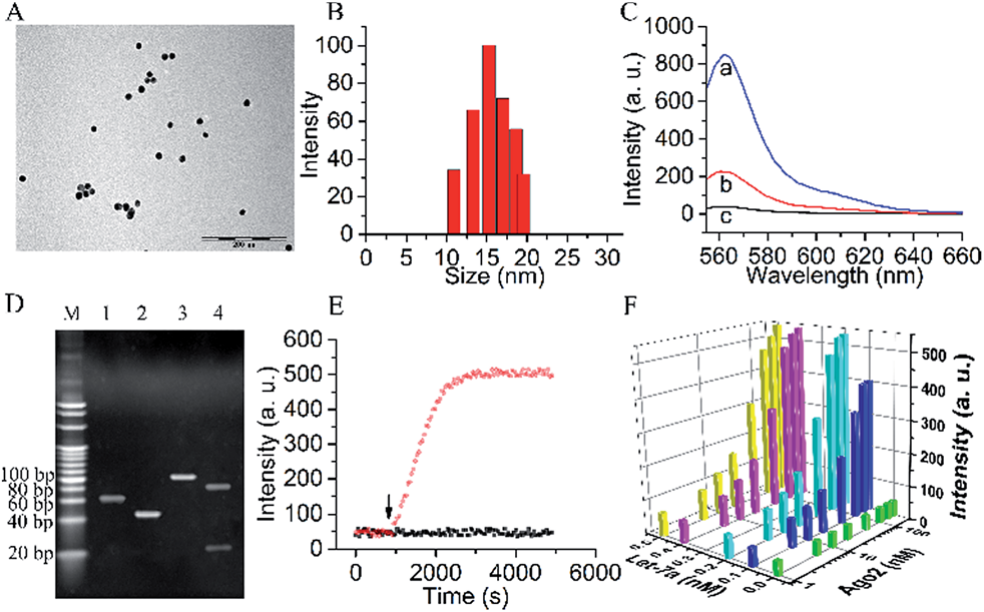
(A) TEM image and (B) DLS of AuNPs. (C) Fluorescence intensities of (a) Strand1 without BHQ2 quencher (Strand1a)/Strand2 duplex in solution; (b) Strand1a/Strand2 duplex on AuNP surface; (c) Strand1/Strand2 duplex on AuNP surface. (D) Nondenaturing polyacrylamide gel electrophoresis (PAGE, 20%). Lane M: marker. Lanes 1–4: (1) Strand1, (2) Strand2, (3) Strand1/Strand2 duplex and (4) Strand1/Strand2 duplex treated with Ago2/Let-7a complex. (E) Time-dependent photobleaching of the probe treated with (black dots) and without (red dots) Ago2/Let-7a complex. The arrow marks the time point of the addition of the Ago2/Let-7a complex. (F) The orthogonal experiments of the Ago2 concentration and Let-7a concentration selection.

A biomimetic probe was designed which shuttled an oligonucleotide duplex into living cells to inhibit the activity of Ago2/Let-7a, the core element of RISC. As shown in Scheme 1, 13 nm gold nanoparticles (AuNPs) (Fig. 1A and B) were chosen as the backbones for the loading of alkylthiol-terminated oligonucleotides and cellular delivery. As a biocompatible material, AuNPs show desirable properties including stability in the cytoplasm, the ability to penetrate through the cell membranes, increased resistance to enzymatic degradation, as well as a large surface area to load oligonucleotides and keep them at close proximity. A DNA strand (Strand1) serving as the frame was immobilized on a AuNP with a thiol group (SH) at the 5′ end. Strand2, designed to recognize target RISC with an RNA fragment, was hybridized with the complementary sequence of Strand1. It should be noted that a spacer of seven nucleotide bases was added between the thiol moiety and the complementary sequence of Strand1 to decrease the steric interference and increase the binding efficiency between the Ago2/Let-7a complex and Strand2. The 5′ end of Strand2 was labeled with a fluorophore (Cy3). In the original state, the fluorescence of Cy3 was quenched by both AuNP (17.68 nm (52 bp) between Cy3 and Au) and BHQ2 (Fig. 1C). In the presence of the Ago2/Let-7a complex, Strand2 was cleaved (Table S1†), resulting in a shorter oligo part with a corresponding lower melting temperature (23.7 °C) than that of the original full-length Strand2 (61.9 °C). The shorter oligonucleotide strand containing the Cy3 fluorophore was released with a recovered fluorescence signal, indicating successful cleavage by the RISC complex. Cyclic cleavage of Strand2 was initiated *via* the released Ago2/Let-7a complex, resulting in an increased fluorescence intensity along with time. Characterization of the probe, including UV-vis spectra, zeta potential and fluorescence, has been carried out to prove the successful synthesis of the desired structure, and a detailed description is shown in the ESI (Fig. S1†). Based on calculations (Fig. S1C and eqn (2)†), about 90 copies of the Strand1/Strand2 duplex were modified on the surface of each AuNP. The highly dense package of oligonucleotides on the probe protects endoribonuclease against proteinase attack.^20,21^

To verify the Ago2/Let-7a complex cleavage reaction, the products of the reaction were tested using nondenaturing polyacrylamide gel electrophoresis (PAGE) (Fig. 1D). As expected, after incubation with the duplex of Strand1/Strand2, a well-defined band of the shorter oligonucleotide strand (22 nt) containing the Cy3 fluorophore was observed in the presence of the Ago2/Let-7a complex (lane 4). In contrast, no distinguishable band was observed in the negative control without the Ago2/Let-7a complex (lane 3). The photostabilities of the fluorophores labelled on the probe before and after cleavage with the Ago2/Let-7a complex were evaluated at the fluorescence maximum wavelength (*λ*_em_ = 562 nm) upon irradiation with a Xe lamp. As shown in Fig. 1E, without addition of the Ago2/Let-7a complex, the fluorescence intensity was constant at a low level (red dots). After treatment with the Ago2/Let-7a complex (150 nM Ago2 and 0.2 nM Let-7a), the fluorescence was turned on and then remained stable after 35 min irradiation, suggesting great photostability under light irradiation. The orthogonal experiments design (OED) method was applied to study the influence of concentration on the cleavage efficiency of the Ago2/Let-7a complex towards the biomimetic probe. As shown in Fig. 1F, the fluorescence intensities increased with the increase of Ago2 concentration and Let-7a concentration, and reached saturation with 150 nM Ago2 and 0.2 nM Let-7a. The saturated reaction time of Ago2 and Let-7a was optimized as 10 min, while the incubation time for the biomimetic probe with the Ago2/Let-7a complex was optimized as 30 min (Fig. S2†).

Monitoring RISC facilitates the investigation of the RNAi pathway. With the proposed fluorophore labelled biomimetic probe, down to 0.2 nM Ago2 and 6.39 pM Let-7a could be quantified in a standard sample (Fig. S3†). The sensitivity for Ago2 is comparable to the western blot method used for the assay in cell extract. The detection limit of Let-7a enables the intracellular analysis of Let-7a down to ∼nM levels in cancer cells.^22–24^ Therefore, the biomimetic probe could be used to monitor RISC of Ago2/Let-7a in living cells. In addition, the probe showed good selectivity to discriminate the Ago2/Let-7a complex (Fig. S4†). Herein, we adopted the A549 lung cancer cell line as the model. The Let-7a concentration in A549 cells was calculated as 0.23 nM using a quantitative reverse transcriptase polymerase chain reaction (qRT-PCR) method and scanning ion conductance microscopy (SICM) technology (Fig. S5†), which reached the saturated concentration from the orthogonal experiments (Fig. 1F). Ago2 in the A549 cell extract was then quantified using the probe with a concentration of 21.4 ± 1.2 nM. With additions of 0–50 nM Ago2 in the cell extracts, recoveries of 100.6–115.9% were achieved (Table S2†). Therefore, intracellular monitoring and controlling the Ago2/Let-7a complex could proceed *via* the biomimetic probe for the regulation of gene expression.

**Fig. 2:**
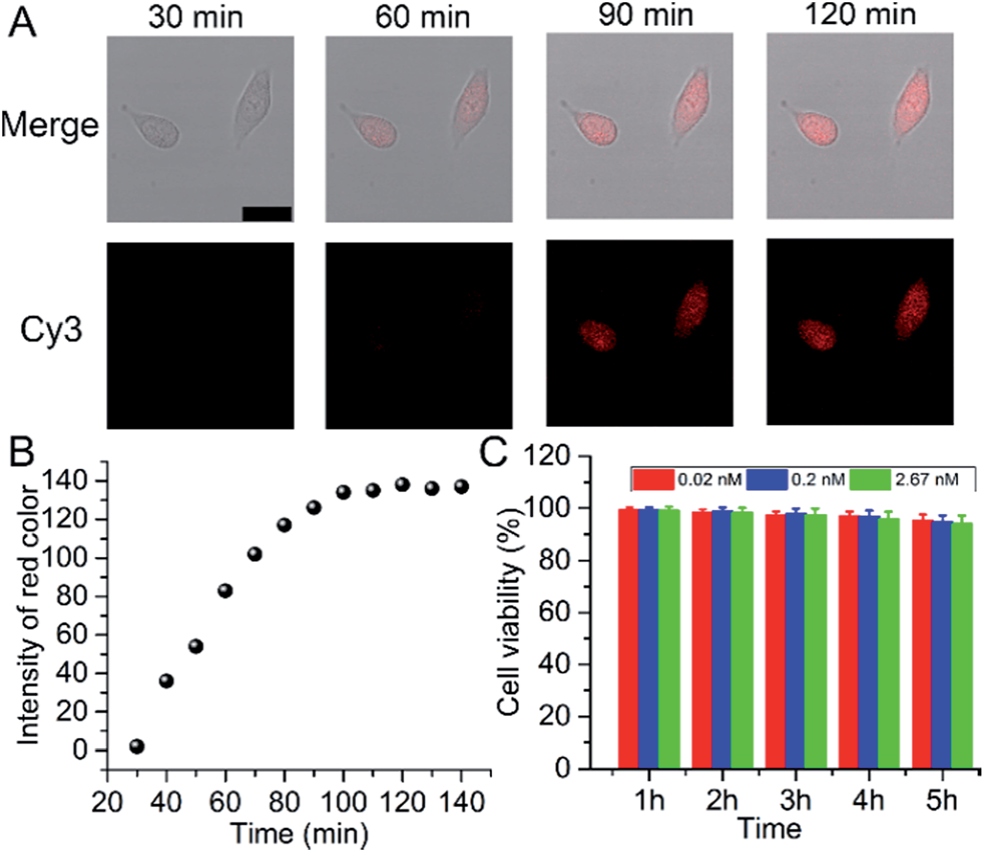
(A) Time course of confocal images of A549 cells incubated with 25 μL of probe (scale bar 25 μm). (B) Plots of fluorescence intensity obtained by Photoshop software *vs.* incubation time in the cell area. (C) Viability of A549 cells treated with the probe at concentrations of 0.02, 0.2, and 2.67 nM from 1 to 5 hours.

In 2013, Asanuma’s group reported a strategy for the RISC assay using a siRNA carrying a fluorophore–quencher pair for the selective labeling of mature RISC with high sensitivity and selectivity, but a relatively long time (24 hours) for detection.^25^ Herein, for intracellular imaging gene regulation of RISC, A549 cells were seeded in a 20 mm confocal dish and sent for confocal observation after the addition of 25 μL of probe. Within the initial 30 min, no obvious fluorescence signal was observed. After 60 min, a fluorescence emission occurred in the living cells, indicating the presence of RISC and the cleavage of Strand2. The fluorescence intensity gradually increased with the increasing incubation time and reached a plateau after 90 min (Fig. 2A), which was much faster than the report. This could be ascribed to the gold nanoparticles used as the backbones of the probes, which facilitated the cellular delivery and made the monitoring of RISC more efficient. RGB (red/green/blue) analysis was adopted for the quantification of the fluorescence intensity of Cy3 in the cell area using Adobe Photoshop software (intensity of red color), and the corresponding results are shown in Fig. 2B. The cytotoxicity of the probe was tested by an MTT (3-(4,5-dimethylthiazol-2-yl)-2,5-diphenyltetrazolium bromide) assay in A549 cells as an example. About 93.9% of the cells remained viable after incubation with 2.67 nM probe, even after 5 h incubation (Fig. 2C), which confirmed that the probe was adequate for intracellular assays. Under the optimal incubation time of 90 min, the relative expression levels of the RISC in the A549, human prostatic carcinoma (PC-3), and human breast cancer (MCF-7) cell lines were determined. Inductively coupled plasma atomic emission spectroscopy (ICP-AES) was used to analyze the cellular uptake of the probe (Fig. S6†). Different types of cells showed uptake of similar amounts of the probe (about 3500) after they were incubated with the probe, excluding the contribution of the difference in the probe uptake to the fluorescence signal. Compared to PC-3 and MCF-7 cells, A549 cells showed the highest expression of RISC (Fig. S7†).

The synthesis strategy for the RISC targeting probe is universal. To prove it, a probe targeting another RISC complex, Ago2/miR-21, was synthesized and employed to monitor the corresponding RISC (Ago2/miR-21 complex combined with Dicer and TRBP) in HeLa and HEK293 cells. The uptake amounts of the probe were also close to 3500 (Fig. S6†). As shown in Fig. S8,† intracellular experiments proved the successful synthesis and excellent specificity of the probe. The universality of the proposed strategy facilitates the study of different RNAi pathways and gene regulation in many kinds of cells.

**Fig. 3:**
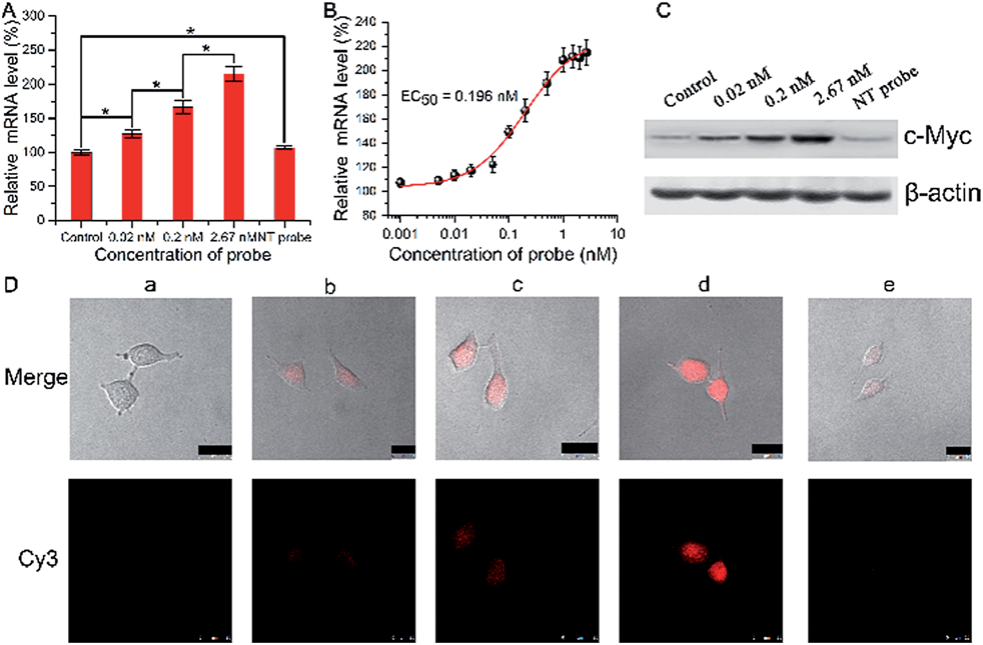
(A) qRT-PCR analyses of c-Myc mRNA expression in the A549 cells treated with biomimetic probes or NT probe (non-target probe). (B) mRNA levels *vs.* the incubated probe concentrations. (C) Western blot analyses of c-Myc protein expression in A549 cells after treatment with biomimetic probes or NT probe, probed with anti-c-Myc antibodies and anti-β-actin antibodies. (D) Confocal images of A549 cells: without (a) and with treatment with: (b) 0.02 nM, (c) 0.2 nM, and (d) 2.67 nM probe, and (e) 2.67 nM NT probe (scale bar 25 μm).

We then investigated and imaged the gene regulation using the Ago2/Let-7a targeting probe. Since Let-7a down-regulates the expression of c-Myc, we monitored the c-Myc expression level in A549 cells after incubation with the biomimetic probe. After incubation for 1.5 h with different concentrations of the probe and a non-target probe (NT probe), which was prepared using a Strand1/Strand2a (random genic mutations in the green part of DNA2) duplex, followed by incubation with fresh growth medium for another 48 h, qRT-PCR analysis showed a mild increase in the mRNA level (Fig. 3A) along with the increase of the probe concentration. The mRNA levels also show a clear concordant effect with the incubated probe concentrations with an EC_50_ (concentration for 50% of maximal effect) value of 0.196 nM in these assays (Fig. 3B). Western blot assays showed consistent results with qRT-PCR at the c-Myc protein level (Fig. 3C). A quite significant increase in the protein level was exhibited in a dose-dependent manner. Conversely, the cells treated with the NT probe showed no obvious changes in both mRNA and protein levels. As shown in confocal images (Fig. 3D), the fluorescence of Cy3 became stronger when the concentration of biomimetic probes increased, indicating that more RISCs were seized by the probes, which should disturb the Let-7a leading RNAi pathway. The mRNA and c-Myc protein levels evaluated by qRT-PCR and western blot were correlated with the fluorescence intensities from confocal images, which reflected the competitive efficiency of RISC by the biomimetic probes. Based on such results, we confirmed the successful cellular function of the biomimetic probe for the c-Myc gene expression regulation and *in situ* imaging, and raised the highly possible mechanism that Let-7a regulated c-Myc gene expression *via* the RNAi pathway.

**Fig. 4:**
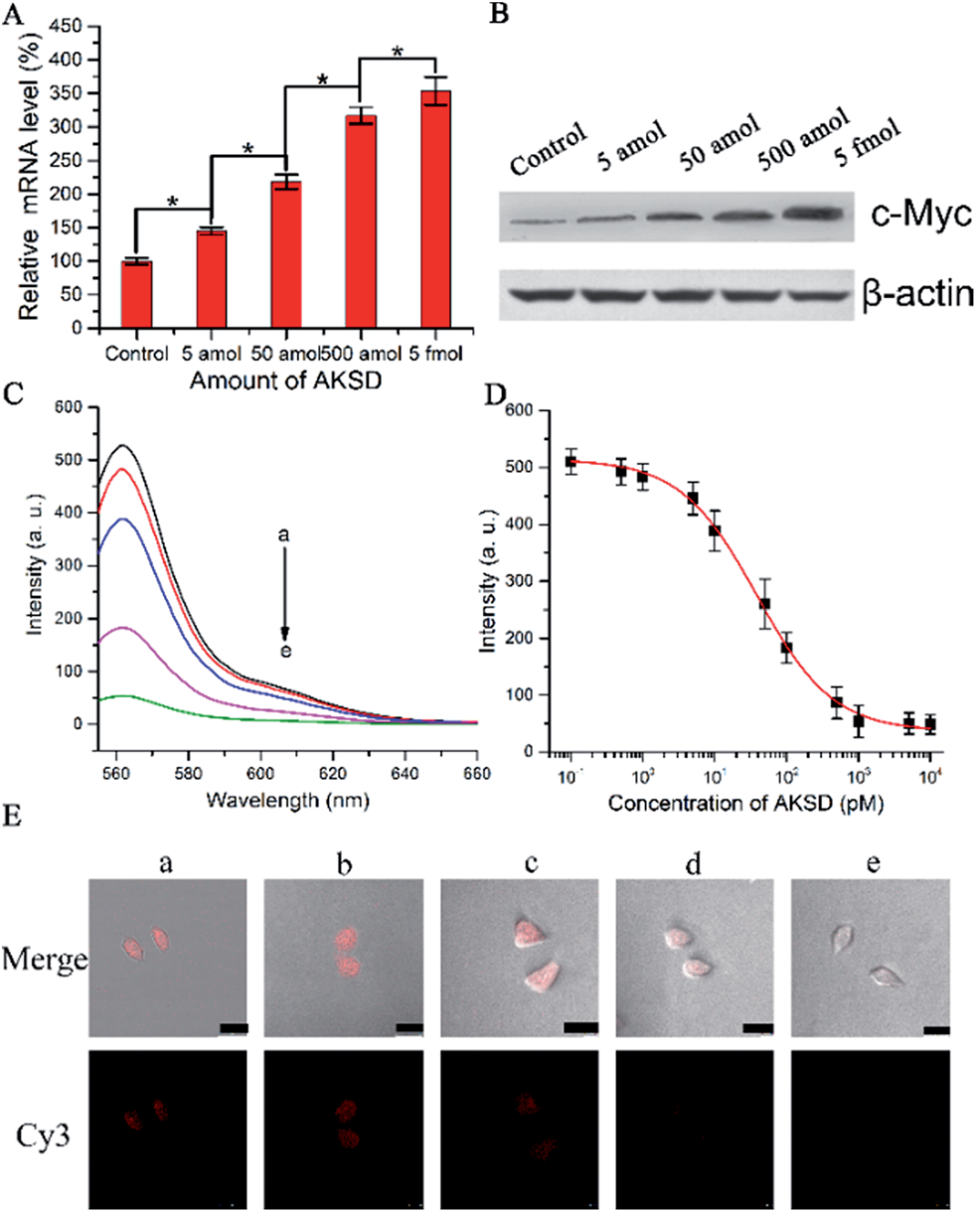
(A) qRT-PCR analyses of c-Myc mRNA expression in the A549 cells treated with AKSD or control (without AKSD). (B) Western blot analyses of c-Myc protein expression in the A549 cells after treatment with AKSD or control, probed with anti-c-Myc antibodies and anti-β-actin antibodies. (C) Fluorescence spectra of the buffer with Ago2 (150 nM)/Let-7a (0.2 nM) complexes treated with AKSD from (a) to (e): 0, 1, 10, 100, and 1000 pM. (D) Fluorescence intensity *vs.* concentration of AKSD. (E) Confocal images of A549 cells: without (a) and with treatment with AKSD: (b) 5 amol, (c) 50 amol, (d) 500 amol, and (e) 5 fmol; then incubated with 25 μL of probe for 90 min (scale bar 25 μm).

To determine whether the biomimetic probe is capable of regulating and imaging intracellular c-Myc levels within living cells by the action of the RNAi pathway, siRNA duplex (Ago2 knockdown siRNA duplex, AKSD, Table S1†) was introduced to knock down Ago2 in A549 cells. The qRT-PCR results shown in Fig. 4A indicated that AKSD induced a dramatic increase of the c-Myc mRNA level in a dose-dependent manner. Western blot analysis showed consistent results with qRT-PCR at the protein level (Fig. 4B). These data indicate that intracellular RISC regulates c-Myc gene expression directly. Before evaluating the probe’s ability to image gene regulation with varying intracellular RISC activity under treatment with AKSD, we firstly added AKSD in a standard sample of Ago2/Let-7a (150 nM : 0.2 nM) with 25 μL biomimetic probe *in vitro* to certify that the probe can also react with RISC even in the presence of AKSD. As shown in Fig. 4C and D, with the increase of AKSD concentration, the fluorescence intensity decreased, suggesting that the probe possesses specificity for RISC. The variation of intracellular RISC complex activity during treatment with AKSD was also monitored by observation. After incubation with the AKSD using Lipofectamine 3000 as a carrier, 25 μL of probe was added to the A549 culture dish for 1.5 h incubation before the collection of confocal images. As the amount of AKSD increased, the fluorescence intensity of the treated cancer cells became weaker (Fig. 4E). Combined with the data in Fig. 4A and B, we arrived at the conclusion that the c-Myc gene is regulated by RISC through the RNAi pathway. Although siRNA-based methods for perturbing endogenous gene regulation pathways mediated by endogenous microRNAs are currently being evaluated in clinical trials, they rely on cellular machinery to take effect, which results in potential side effects. The RISC targeting probe, on the other hand, works as intracellular machinery without cytokine activation. Furthermore, since the probe can be used for noninvasive monitoring of intracellular RISC activity after Ago2 activity is knocked down, it may serve as a potential tool for the screening of RNA-silencing-related drugs.

## Conclusions

In conclusion, we developed a novel target selective, specific self-delivered, and non-cytotoxic fluorescence biomimetic probe for gene expression regulation and *in situ* imaging in living cells by the action of the RNAi pathway. The practicality of the probe has been demonstrated by dynamically monitoring the gene regulation levels in A549 cells. *Via* disturbing the gene silencing pathway, the c-Myc expression level in A549 cells was up-regulated using the RISC targeting probe. The synthetic method for the biomimetic probe is universally applicable, and could target specific RISC in living cells for the fundamental study of RNAi pathways, or for developing a gene regulation strategy.
