# Supplementary material for: Regulation and imaging of gene expression via an RNA interference antagonistic biomimetic probe
Source: Chem Sci. 2017 May 5;8(7):4973–7. doi: 10.1039/c7sc00909g (PMC5607853; doi:10.1039/c7sc00909g)
Supplement: Supplementary file 1 [file SC-008-C7SC00909G-s001.pdf]

Electronic Supplementary Material (ESI) for Chemical Science.
This journal is © The Royal Society of Chemistry 2017

Supplementary information for:

Regulation and Imaging of Gene Expression via a RNA Interference Antagonistic Biomimetic Probe

Kai Zhang,[a, b] Xue-Jiao Yang,[a] Wei Zhao,*[, a] Ming-Chen Xu,[a] Jing-Juan Xu*[, a] and Hong-Yuan Chen[a]

[a]State Key Laboratory of Analytical Chemistry for Life Science and Collaborative Innovation Center of Chemistry for Life Sciences, School of Chemistry and Chemical Engineering, Nanjing University, Nanjing 210023, China.

[b]Key Laboratory of Nuclear Medicine, Ministry of Health, Jiangsu Key Laboratory of Molecular Nuclear Medicine, Jiangsu Institute of Nuclear Medicine, Wuxi, Jiangsu 214063, China

*Corresponding Author: Phone/fax: +86-25-89687294. E-mail: weizhao@nju.edu.cn (W. Zhao); xujj@nju.edu.cn (J.J. Xu).

Table of contents

1. EXPERIMENTAL SECTION . . . *3*
1.1 Materials and reagents. . . . 3
1.2 Preparation of Strand1/Strand2 functionalized probe. . . . 5
1.3 Evaluation of amount of Strand1/Strand2 duplex assembled on the probe. . . . 6
1.4 Polyacrylamide hydrogel electrophoresis. . . . 7
1.5 Cell culture. . . . 7
1.6 Ago2 knockdown.. . . . 8
1.7 Cytotoxicity of the probe on A549 cells. . . . 8
1.8 RISC Assay in Cell Extract. . . . 9
1.9 Determination Let-7a concentration in A549 single cells. . . . 9
1.10 RNA extraction and qRT-PCR analysis for the RNA levels of c-Myc.. . . . 11
1.11 Western blot analysis of c-Myc proteins. . . . 12
1.12 *In Situ* imaging of RISC with the probe. . . . 12
1.13 The feasibility and universality of the strategy assay. . . . 13
1.14 Apparatus.. . . . 13
2. ADDITIONAL RESULTS AND DISCUSSION . . . *14*
2.1 Quantitative and specific measurement of Ago2 and Let-7a concentrations in buffer solution using the biomimetic probe . . . 14
2.2 Regular monitoring of Ago2/Let-7a complex activity in cell extracts . . . 18
3. ADDITIONAL FIGURES . . . *22*
4. REFERENCES . . . *25*

1. EXPERIMENTAL SECTION

1.1 Materials and reagents.

Table S1. Oligonucleotide sequences used in this strategy

note	sequence (5'-3')
Strand1	SH-AAAAGCACACGTCCATCTCT*TAATCTGCATGCTAGCCATATCGACTATCCG*AATG TGAGA-BHQ2
Strand1a	SH-AAAAGCACACGTCCATCTCT*TAATCTGCATGCTAGCCATATCGACTATCCG*AATG TGAGA
Strand2	Cy3-TCTCACATTAACUAUACAACC↓UACUACCUCAAAGAGATGGACGTG
Strand2a	Cy3-TCTCACATTAATUAUGCAAGCUAGUACTUCAAAGAGATGGACGTG
Let-7a	UGAGGUAGUAGGUUGUAUAGUU
Let-7b	UGAGGUAGUAGGUUGUGUGGUU
Let-7c	UGAGGUAGUAGGUUGUAUGGUU
Strand3	Cy3-TCTCACATTAUCAACAUCAG↓UCUGAUAAGCUAAAGAGATGGACGTG
miR-21	UAGCUUAUCAGACUGAUGUUGA
AKSD1	CGAUCGGCAAGAAGAGAUUAG
AKSD2	AAUCUCUUCUUGCCGAUCGGG
Let-7a Fw	TGAGGTAGTAGGTTGTATAGTT
c-Myc Fw	GCCACGTCTCCACACATCAG
c-Myc Rv	TGGTGCATTTTCGGTTGTTG
GAPDH Fw	GGTCTCCTCTGACTTCAACA
GAPDH Rv	AGCCAAATTCGTTGTCATAC

The sequences shown in underline of Strand1 or Strand1a are complementary to the underlined sequences that shown in Strand2 (or Strand2a) and Strand3. The sequences shown in green color of Strand2 (or Strand2a) and Strand3, and yellow colors of Let-7a, Let-7b, and Let-7c are constituted by ribonucleic acid bases. The red letters in Strand2a symbolize the mutant bases in the Strand2a and the red letters in Let-7b, and Let-7c symbolize the mutant bases in the Let-7a. The arrow in Strand2 marked the cleavage point of Ago2/Let-7a complex and the arrow in Strand3 marked the cleavage point of Ago2/miR-21 complex. The colors of the sequences are the same as given in Scheme 1.

Chloroauric acid ($HAuCl_4•4H_2O$) was obtained from Shanghai Chemical Reagent Company (Shanghai, China). Argonaute1, argonaute2, and argonaute3 were purchased from Sino Biological Inc (Beijing, China). Trisodium citrate was obtained from Sinopharm Chemical Reagent Co., Ltd. (Shanghai, China). Tris (2-carboxyethyl) phosphine hydrochloride (TCEP) and Diethyl pyrocarbonate (DEPC) were purchased from Sigma-Aldrich Inc. (St. Louis, Missouri, USA). DNase I endonuclease, A549 cells, PC-3 cells, MCF-7 cells, HeLa cells and HEK293 cells were from KeyGen Biotech. Co. Ltd. (Nanjing, China). RNasin, Gel electrophoresis loading buffer and ladder DNA were purchased from Takara Biotech. Co. Ltd. (Dalian, China). Aqua regia was prepared by mixing HCl and HNO_3 with the volume ratio of 3:1. All other reagents were of analytical grade. All aqueous solutions were prepared using ultrapure water (> 18 MΩ cm, Milli-Q, Millipore). The strand sequences were purchased from Genscript Biotech. Co., Ltd. (Nanjing, China) with the sequences as shown in Table S1.

In order to create and maintain an RNase-free environment, all solutions in this work were treated with 0.1% DEPC and autoclaved. The tips and tubes are RNase-free and do not require pretreatment to inactivate RNases. The buffers used in this work were prepared as follows: Buffer A: Phosphate buffer saline (PBS, pH 7.4) contained 136.7 mM NaCl, 2.7 mM KCl, 8.72 mM Na_2HPO_4, 1.41 mM KH_2PO_4, and 10 mM TCEP. Buffer B: Phosphate buffer saline (PBS, pH 7.4) contained 136.7 mM NaCl, 2.7 mM KCl, 8.72 mM Na_2HPO_4, 1.41 mM KH_2PO_4, 0.1% DEPC, and 1 U μL^{-1} RNasin.

1.2 Preparation of Strand1/Strand2 functionalized probe.

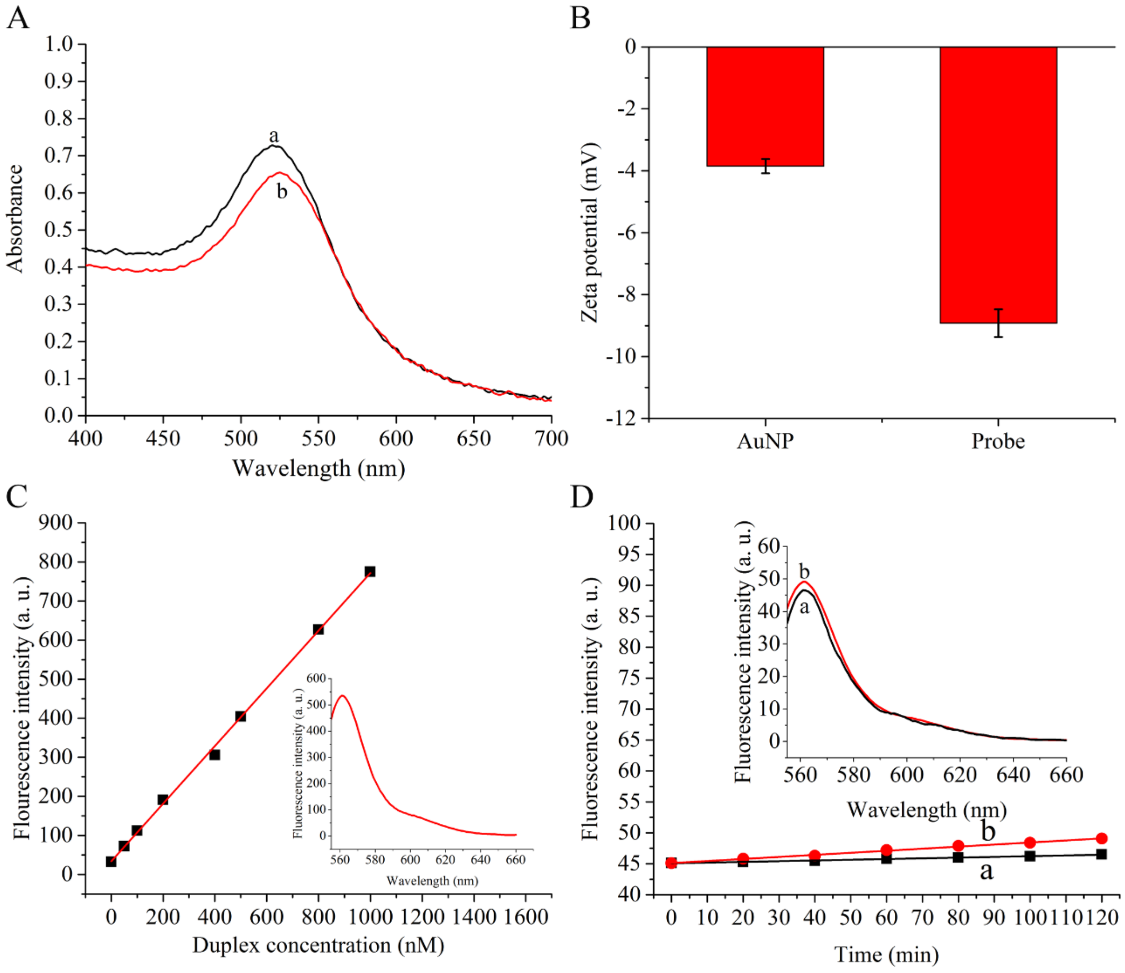

Fig. S1 (A) UV−vis spectra of (a) AuNPs and (b) the probe. The data of ε_{520} (2.7×10^8 M^{-1} cm^{-1}) [1] can be used to calculate the particle concentration c in mol per litre from the absorption A at 520 nm (0.72) for a standard path length l of 1 cm. (B) Zeta potentials of AuNPs and the probe. (C) Plot of fluorescence intensity vs. Duplex concentration. Inset: fluorescence spectrum of the supernatant containing excess Strand2 collected after the preparation of probe. (D) Plots of fluorescence intensity of the probe in absence (a) and presence (b) of 1 U mL^{-1} DNase I vs. incubation time. Inset: fluorescence spectra corresponding to a and b at 120 min.

Gold Nanoparticles (AuNPs) were prepared according to the previous report.[2] After heating 200 mL $HAuCl_4$ solution (0.01%) to 100 °C, 5.0 mL trisodium citrate (1%) was added quickly to the boiling solution under continuous stirring. The reaction mixture was stirred at 100 °C for 1 h until the color turned deep red and then stored at 4 °C. The diameter of such prepared nanoparticles is ~13 nm (Fig. 1A, and Fig. 1B). The extinction value of the 520 nm plasmon peak is ~ 0.72 (Fig. S1A), and the nanoparticle

concentration is ~ 2.67 nM calculated by the followed equation (equation 1):

$$c = \frac{A_{520}}{\varepsilon_{520}}$$

In which, extinction coefficient at λ_{520} (ε_{520}) is 2.7×10^8 M^{-1} cm^{-1} for 13 nm particles.

To reduce the disulfide linkage of the thiol-modified Strand1, 10 μL of 100 μM Strand1 was mixed with 1.5 μL Buffer A. After 1 h., the reduced Strand1 and 10 μL Strand2 (100 μM) which dissolved by Buffer B were added to 1 mL of AuNP solution in a clean glass vial while gently shaking by hand. The vial was stirred at room temperature overnight. Afterward, 0.1 mL PBS solution containing 2 M NaCl was added to the mixture stepwise for stabilizing the obtained probe, which was centrifuged and washed with Buffer B twice, finally resuspensed in 1 mL Buffer B. The supernatant containing excess duplex was collected for determining the amount of duplex on each probe.

1.3 Evaluation of amount of Strand1/Strand2 duplex assembled on the probe.

For quantifying the oligonucleotides loaded on each AuNP, we employed Strand1a which has the same sequences as Strand1 but without modification of BHQ2 from the 3' end. The fluorescence intensity of series concentration of Strand1a/Strand2 duplex was detected to establish a calibration curve. Slit widths for excitation and emission were 3 nm and 3 nm in order to fit the upper limit of the measurement range, respectively. As shown in Fig. S1C, as the concentration of duplex increased, the intensity was enhanced, a linear range from 0 to 1000 nM was observed with Y= 0.738X+33.87. After modification process, the intensity of duplex retained in supernatant was 534.74 (Fig. S1C, insert) and the concentration was 678.69 nM calculated by the above linear equation. As the total concentration of duplex used in the process was 892.46 nM (C= $\frac{C_{origin} \times V_{origin}}{V_{total}}$, where C_{origin} is 100 μM, V_{origin} is 10 μL, and V_{total} is 1120.5 μL (1000 μL+10 μL+1.5 μL+10 μL+100 μL)), then the duplex amount loaded on one AuNP was estimated to be 90 by the followed equation (equation

2):

$$N = \frac{N_{duplex}}{N_{Au}} = \frac{N_{total} - N_{solution}}{N_{Au}} = \frac{n_{total} \times N_A - n_{solution} \times N_A}{n_{Au} \times N_A}$$
$$= \frac{(n_{total} - n_{solution}) \times N_A}{n_{Au} \times N_A}$$
$$= \frac{(C_{total} \times V_{total} - C_{solution} \times V_{solution}) \times N_A}{C_{Au} \times V_{Au} \times N_A}$$
$$= \frac{(C_{total} \times V_{total} - C_{solution} \times V_{total}) \times N_A}{C_{Au} \times V_{Au} \times N_A} \quad *$$
$$= \frac{(C_{total} - C_{solution}) \times V_{total} \times N_A}{C_{Au} \times V_{Au} \times N_A} = \frac{(C_{total} - C_{solution}) \times V_{total}}{C_{Au} \times V_{Au}}$$

* $V_{solution}$ approximately equal to V_{total}

Where C_{total} is the total concentration of duplex (892.46 nM), $C_{solution}$ is the duplex concentration of the collected supernatant (678.69 nM), V_{total} is the total volume (1120.5 μL), C_{Au} is the original concentration of AuNP (2.67 nM) and V_{Au} is the original volume of AuNP (1000 μL). Since the amount of Strand1a/Strand2 on each probe was estimated to be around 90. So, the amount of strand1/strand2 duplex on the well-designed probe was also to be around 90.

1.4 Polyacrylamide hydrogel electrophoresis.

Nondenaturing polyacrylamide gel electrophoresis (PAGE) (20%) was carried out in 1× TBE buffer at a 120 V constant voltage for 1.5 h at room temperature. Before cleavage reaction, the mixture of Strand1 and Strand2 in Buffer B was heated to 90 °C for 10 min and allowed to cool to room temperature for about 4 h to form the partially complementary ds-strands. Then the above solution was incubated with Ago2/Let-7a complex (150 nM Ago2 incubated with 0.2 nM Let-7a) at 37 °C for 30 min. The gel was taken photograph under UV light after staining with GelRed for 15 min. The concentration of each strand is 1 μM.

1.5 Cell culture.

A549 cells and MCF-7 cells were respectively cultured in a flask in Dulbecco's modified Eagle's medium (RPMI-1640, GIBCO) supplemented with 10% fetal

calf serum (FCS, Sigma), penicillin (100 μg mL^{-1}), and streptomycin (100 μg mL^{-1}) at 37 °C in a humidified atmosphere containing 5% CO_2. PC-3 was cultured in F12k (GIBCO) supplemented with 10% fetal calf serum, penicillin (100 μg mL^{-1}), and streptomycin (100 μg mL^{-1}) at 37 °C in a humidified atmosphere containing 5% CO_2. HeLa and HEK293 cells were respectively cultured in a flask in DMEM (GIBCO) supplemented with 10% fetal calf serum (FCS, Sigma), penicillin (100 μg mL^{-1}), and streptomycin (100 μg mL^{-1}) at 37 °C in a humidified atmosphere containing 5% CO_2. Cell number was determined with a Petroff-Hausser cell counter (USA).

1.6 Ago2 knockdown.

Ago2 knockdown in A549 cell lines was performed by transfecting a siRNA duplex (AKSD1 and AKSD2, Table S1) using Lipofectamine 3000 (Invitrogen, Grand Island, NY, USA) following the manufacturer's instructions. Briefly, 24 h prior to transfection, the cells were seeded in a culture dish at a cell density of 0.5×10^6 cells. Then, 5 μL siRNA duplex with different amounts of substance (0, 5 amol, 50 amol, 500 amol, and 5 fmol) were mixed in 125 μL Opti-MEM Medium reduced serum media and then 4 μL Lipofectamine 3000 was diluted in 125 μL Opti-MEM Medium reduced serum media. Then added diluted siRNA duplex to diluted Lipofectamine 3000 and incubated at room temperature for 15 minutes. After the incubation, added the mixture to the cells and incubated the cells for 48 h.

1.7 Cytotoxicity of the probe on A549 cells.

A549 cells were dispensed into 96-well plates at a final concentration of 1×10^4 cells per well in a culture medium (100 μL), and incubated overnight before treatment. The culture medium was then removed and replaced with new medium

containing the probe at varying concentrations of 0.02, 0.2 and 2.67 nM for 1.5 h. The solution was then replaced with fresh medium and the cell viability was measured using the MTT (3-(4,5-dimethylthiazol-2-yl)-2,5-diphenyltetrazolium bromide) assay after 48 h incubation.

1.8 RISC Assay in Cell Extract.

Cells were collected in the exponential phase of growth, and 5×10^7 cells were dispensed in a 1.5 mL EP tube, washed twice with ice-cold PBS (0.1 M, pH 7.4), and resuspended in 200 μL of ice-cold CHAPS lysis buffer containing 10 mM Tris-HCl (pH 7.5), 1 mM $MgCl_2$, 1 mM EGTA, 0.1 mM PMSF, 0.5% CHAPS and 10% glycerol. The mixture was incubated for 30 min on ice and centrifuged at 16000 rpm and 4 °C for 20 min. The supernatant was collected and diluted to 200 μL as cell extract for detection or storage at -80 °C.

After incubating the mixtures of 10 μL cell extract (corresponding to 5×10^5 cells), and 100 μL probe in the absence and presence of 10 μL Ago2/Let-7a complex (different concentrations of Ago2 incubated with 0.2 nM Let-7a) at 37 °C for 1 h, the fluorescent intensity was recorded, respectively.

1.9 Determination Let-7a concentration in A549 single cells.

1.9.1 Quantitative reverse transcriptase polymerase chain reaction (qRT-PCR) analysis of siRNA inside cells.

Total RNA was extracted from A549 cells using Trizol reagent (Invitrogen, Beijing, China) following the manufacturer's instructions. The integrity of total RNA was assessed by gel electrophoresis. The poly (A) tails were added to the extracted siRNAs by *E. coli* Poly (A) polymerase (Fermentas, USA). The cDNA samples were then

prepared using Hifi-MMLV first Strand cDNA Synthesis Kit (CWbio. CO. Ltd, Beijing, China). Briefly, a total volume of 20 μL solution containing 8 μL of the poly (A) reaction mixture, 3 μL upstream primer (25 μM), 1 μL dNTPs (10 mM for each of dATP, dGTP, dCTP and dTTP), 4 μL 5 × SuperRT Reverse Transcriptase buffer, 0.5 μL Super RT Reverse Transcriptase (200 U μL^{-1}, CWbio.CO. Ltd, Beijing, China) and 3.5 μL nuclease-free water was incubated at 42 °C for 50 min followed by heat inactivation of reverse transcriptase for 5 min at 85 °C. The cDNA samples were store at -20 °C for future use. Upstream primer used for reverse transcription reaction of Let-7a was given in table S1 (Let-7a Fw).

qRT-PCR analysis of siRNA was performed with siRNA Real-Time PCR Assay Kit (CWbio. CO. Ltd, Beijing, China) according to the manufacturer's instructions on an ABI 7500 Real-Time PCR instrument (Applied Biosystems, USA). The 20 μL reaction solution contained 2 μL of cDNA sample (The amount of cDNA is equal to the amount of Let-7a obtained from 100 A549 cells), 10 μL 2 × siRNA qPCR premix (with SYBR and ROX), 0.5 μL upstream primer (10 μM), 0.5 μL downstream primers (10 μM, provided within siRNA Real-Time PCR Assay Kit) and 7 μL nuclease-free water. The qPCR conditions were as follows: staying at 95 °C for 10 min, then followed by 40 cycles of 95 °C for 15 s and 60 °C for 60 s (ABI7500 sequence detection system (Life Technologies, ON, USA)). C_T values of 25.22 (from curve g of Fig. S5A) were converted into absolute Let-7a copy numbers using a standard curve with a formula of Y=-3.07X+39.02 (Fig. S5B), Where Y is the C_T values from synthetic Let-7a siRNA (Fig. S5A) and X is the logarithm of the copies of Let-7a siRNA. The average number of Let-7a measured from the formula is about 313 copies per cell.

1.9.2 Measure whole cell volume and calculation of the concentration of Let-7a in single-cell.

A549 cells (0.5 mL) of 1×10^{6} mL^{-1} were seeded in culture dish for 24 h, and then were sent for volume measure. Scanning ion conductance microscopy (SICM) was used for

measure the volume of single-cell. The SICM glass nanopipets were pulled in a laser puller (P2000, Sutter Instruments, Novato, CA) to a sharp point with a diameter of 100 nM. The probe is filled with electrolyte (PBS, pH 7.4 contained 136.7 mM NaCl, 2.7 mM KCl, 8.72 mM Na_2HPO_4, and 1.41 mM KH_2PO_4). The Scanning Probe Image Processor (SPIP) software version 6.5.2 (Image Metrology A/S, Denmark) was employed for the simulation of the cells (Fig. S5C and Fig. S5D) and calculation of the volume of the single-cells (by using the path: Analyze-Histogram-Mat. Vol. from the software). The average volume of A549 cells was approximately 2.31 ± 0.17 pL.

1.9.3 The estimation of the concentration of Let-7a in A549 cells.

The average concentration of Let-7a (c_{Let-7a}) in A549 cells was estimated to be 0.23 nM by the followed equation (equation 3):

$$c_{Let-7a} = \frac{n_{Let-7a}}{V_{A549}} = \frac{N_{Let-7a}/N_A}{V_{A549}}$$

Where N_{Let-7a} is the average number of Let-7a in single-cells (313), N_A is Avogadro's number (6.02×10^{23} mol^{-1}), and V_{A549} is the average volume of A549 cells (2.31 pL).

1.10 RNA extraction and qRT-PCR analysis for the RNA levels of c-Myc.

Total RNA samples were extracted from A549 cells using a Trizol reagent (Invitrogen, Beijing, China) according the manufacturer's instructions. To prevent DNA contamination, total RNA was treated with RNase-free DNase II (Invitrogen). Total RNA samples (1 μg per reaction) were reversely transcribed into cDNAs by SuperScript^{TM} First-Strand Synthesis System (Invitrogen). qRT-PCR was then performed with the cDNA using SYBR green PCR Master Mix (Takara, Shiga, Japan). Each amplification reaction underwent denaturation at 95 °C for 30 s, amplification for 40 cycles at 95 °C for 10 s, annealing and extension at 60 °C for 20 s using ABI7500 sequence detection system (Life Technologies, ON, USA). The primers of mRNA forward (c-Myc Fw) and mRNA reverse (c-

Myc Rv) were all listed in Table S1.[4] In all experiments, the human glyceraldehyde-3-phosphate dehydrogenase (GAPDH) was used as an internal control in qRT-PCR amplification, and its forward primer (GAPDH Fw) and reverse primer (GAPDH Rv) were also listed in Table S1.

1.11 Western blot analysis of c-Myc proteins.

Cell lysates were collected from A549 cells in ice-cold lysis buffer (10 mM Tris, 150 mM NaCl, 1 mM EDTA, 0.1 % sodium dodecyl sulfate, 1 % Triton X-100, that contained the protease inhibitors phenylmethylsulfonyl fluoride, 200 mg/mL, and leupeptin, 3 mg/mL, pH 7.4) for 5 min on ice followed by centrifugation at 4 ºC for 15 min to sediment particulate materials. Then protein samples were separated by 12 % SDS-polyacrylamide gel (SDS-PAGE) and transferred to polyvinylidene fluoride (PVDF) membranes. Each membrane was incubated for 1 h in blocking solution containing 5 % bovine serum albumin (BSA) in PBS-Tween at room temperature and then immunoblotted with monoclonal anti-c-Myc antibody (KeyGen Biotech) and β-Actin Monoclonal Antibody (KeyGen Biotech) at 4 °C overnight. The membrane was washed with PBS-Tween and then visualized using the enhanced chemiluminescence (ECL) assay kit (Beyotime, Nantong, China) according to the manufacturer’s directions.

1.12 *In Situ* imaging of RISC with the probe.

0.5 mL A549 cells (or PC-3, MCF-7 cells) of 1×10^6 mL^{-1} were seeded in each confocal dish for 24 h, and 25 μL probe was then added into each cell-adhered dish. After incubation at 37 °C for different times, the cells were sent for fluorescent confocal imaging detection.

1.13 The feasibility and universality of the strategy assay.

To demonstrate the feasibility and universality of the probe, a homogeneous model was adopted for the detection of relative expression levels of the RISC (Ago2/miR-21 complex combined with Dicer and TRBP) in HeLa cells with just few oligonucleotides sequence change in the green part of Strand2, and the sequence of the strand (Strand3) was listed in Table S1. 0.5 mL HeLa (human cervical cancer) cells (or HEK293 (human normal kidney) cells) of 1×10^6 mL^{-1} were seeded in each confocal dish for 24 h, and 25 μL probe was then added into each cell-adhered dish. After incubation at 37 °C for 90 min, the cells were sent for fluorescent confocal imaging detection.

1.14 Apparatus.

The transmission electron microscopic (TEM) images were obtained on a JEM-2100 transmission electron microscope (JEOL Ltd., Japan). Dynamic light scattering (DLS) was obtained on a 90 Plus/BI-MAS equipment (Brook haven, USA). Zeta potential analysis was performed on a Zetasizer (Nano-Z, Malvern, UK). The UV-vis absorption spectra were obtained with a UV-vis spectrophotometer (UV-3600, Shimadzu, Japan). Gel electrophoresis was performed on a Bio-Rad electrophoresis analyser and imaged on the Bio-Rad ChemDoc XRS (USA). The fluorescence spectra were obtained on a spectrofluorophotometer (RF-5301PC, Shimadzu, Japan), unless otherwise specified, the slit widths for excitation and emission were all 5 nm. The cell images were gained on a TCS SP5 laser scanning confocal microscope (Leica, Germany). Inductively coupled plasma atomic emission spectra (ICP-AES) was detected with an ICP-AES instrument (Optima 5300DV, USA). Cell volumes were detected on Scanning ion conductance microscopy (SICM, ICnano2000, Ionscope Ltd, UK).

2. ADDITIONAL RESULTS AND DISCUSSION

2.1 Quantitative and specific measurement of Ago2 and Let-7a concentrations in buffer solution using the biomimetic probe

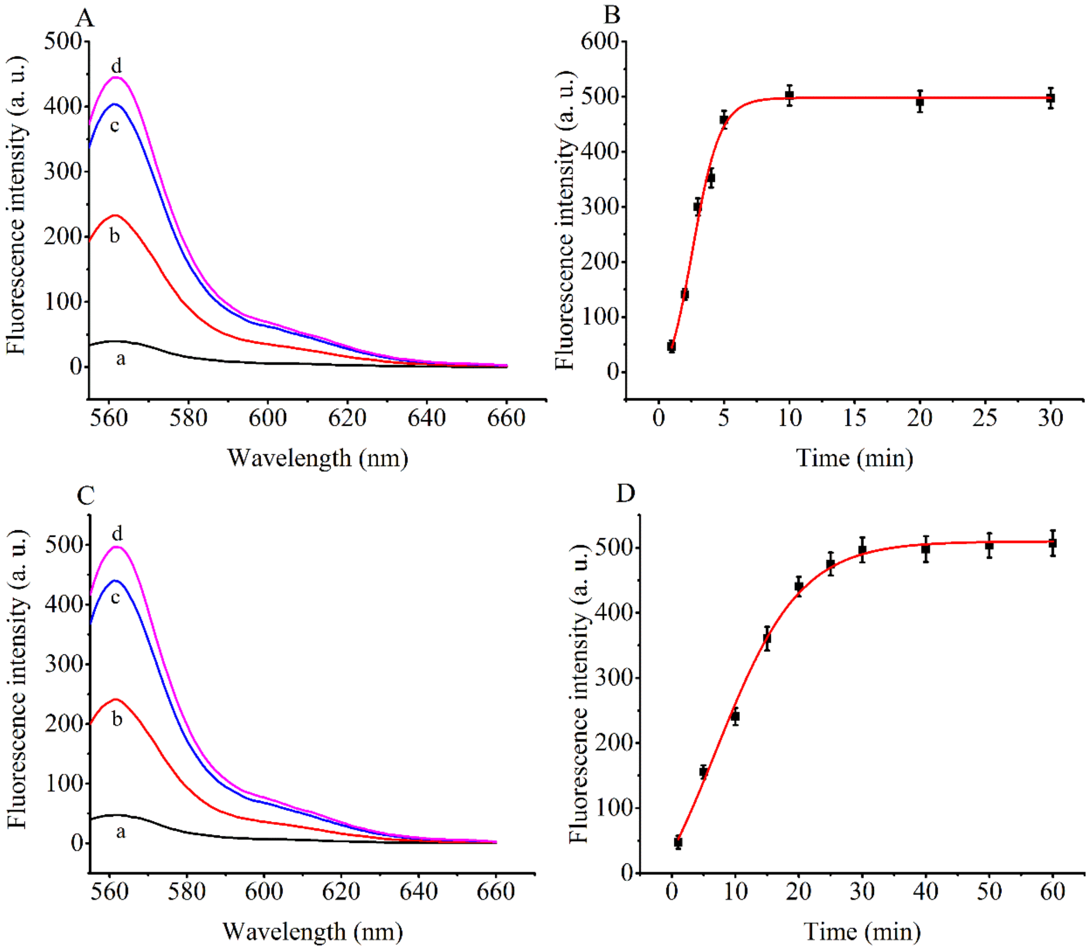

Fig. S2 (A) Fluorescence spectra of the probe after incubation with Ago2 (150 nM) and Let-7a (0.2 nM) complex for 30 min. The Ago2 and Let-7a incubation time were (a) 1, (b) 2, (c) 5, and (d) 10 min, respectively. (B) Plots of fluorescence intensity *vs.* incubation time of Ago2 and Let-7a. (C) Fluorescence spectra of the probe after incubation with Ago2/Let-7a complex (150 nM Ago2 and 0.2 nM Let-7a incubated for 10 min) for (a) 1, (b) 10, (c) 20, and (d) 30 min, respectively. (D) Plots of fluorescence intensity *vs.* incubation time of the probe with Ago2/Let-7a complex. All the data are taken from independent experiments with repetition for at least three times, and the presented data are the results of averaging.

To obtain a perfect assay performance, we have first monitored the kinetics of the Ago2 and Let-7a reaction, and the probe and Ago2/Let-7a complex reaction in phosphate buffered saline, respectively. Firstly, the fluorescence intensity of the probe and the

Ago2/Let-7a complex (150 nM Ago2 and 0.2 nM Let-7a, respectively) was recorded after incubation of 30 min. For the complex formation, the Ago2 and Let-7a incubated with different times. The fluorescence intensity of the mixture increases rapidly with increasing reaction time of Ago2 and Let-7a in the range from 0 to 10 min and reaches a plateau thereafter (Fig. S2A and Fig. S2B). Secondly, in order to test the interaction of probe and Ago2/Let-7a complex (for the formation of Ago2/Let-7a complex, 150 nM Ago2 and 0.2 nM Let-7a were employed and incubated for 10 min before the probe and Ago2/Let-7a interaction), the fluorescence intensity of the mixture of the probe and the Ago/Let-7a complex which incubated with different times in phosphate buffered saline was recorded. The fluorescence intensity of the mixture increases rapidly with increasing reaction time in the range from 0 to 30 min and reaches a plateau thereafter (Fig. S2C, Fig. S2D). These data strongly agree with which list in Fig. 1E (black circles, from the time point of the adding of Ago2/Let-7a complex to the time point of achieving plateau). Hence, to ensure complete reduction reactions, the Ago2 and Let-7a reaction time of 10 min, the probe and Ago2/Let-7a complex reaction time of 30 min were selected for subsequent experiments, respectively.

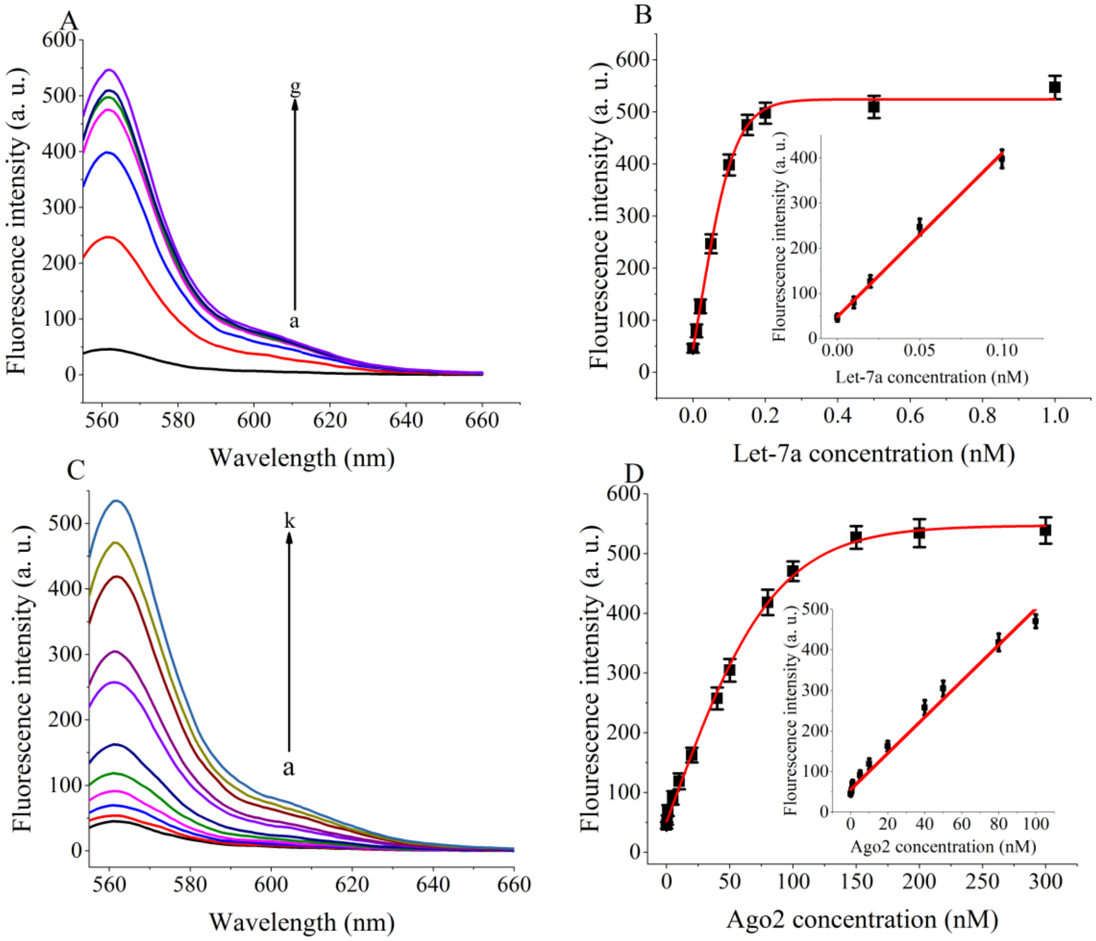

Fig. S3 (A) Fluorescence spectra of the strategy for the investigation of Ago2 (150 nM) with Let-7a at different concentrations: from (a) to (g): 0, 0.05, 0.1, 0.15, 0.2, 0.5 and 1 nM, respectively. (B) Relationship between the fluorescence intensity and the concentration of Let-7a. The inset shows a linear relationship over the concentration range from 0 to 0.1 nM (C) Fluorescence spectra of the strategy for the investigation of Let-7a (0.2 nM) with Ago2 at different concentrations: from (a) to (k): 0, 0.5, 1, 5, 10, 20, 40, 50, 80, 100, and 200 nM, respectively. (D) Relationship between the fluorescence intensity and the concentration of Ago2. The inset shows a linear relationship over the concentration range from 0 to 100 nM. All the data are taken from independent experiments with repetition for at least three times, and the presented data are the results of averaging.

To verify the feasibility of quantitative detection of Ago2 and Let-7a, the fluorescence changes that occur during the emission of Cy3 under different conditions were investigated. Firstly, different concentration of Let-7a and 150 nM Ago2 combined complex were added to the probe solution, and the fluorescence emissions were recorded. As the concentration of Let-7a increased, the fluorescence intensity obviously increased (Fig. S3A). Fig. S3B shows the relationship between the fluorescence intensity and the concentration of Let-7a. A good linear range from 0 to 0.1 nM using

an equation Y= 3626.4 X + 47.87 (R^2 = 0.93), where Y is the fluorescence intensity and X is the concentration of Let-7a, and a detection limit of 6.39 pM could be obtained according to the responses of the blank tests plus 3 times the standard deviation (3σ) (Fig. S3B insert). Then, different concentrations of Ago2 and 0.2 nM Let-7a combined complex were added to the probe solution, and the fluorescence emissions were recorded. As the concentration of Ago2 increased, the fluorescence intensity obviously increased (Fig. S3C). Fig. S3D shows the relationship between the fluorescence intensity and the concentration of Ago2. A good linear range from 0 to 100 nM using an equation Y= 4.45 X + 56.06 (R^2 = 0.982), where Y is the fluorescence intensity and X is the concentration of Ago2, and a detection limit of 0.2 nM could be obtained according to the 3σ method (Fig. S3D insert).

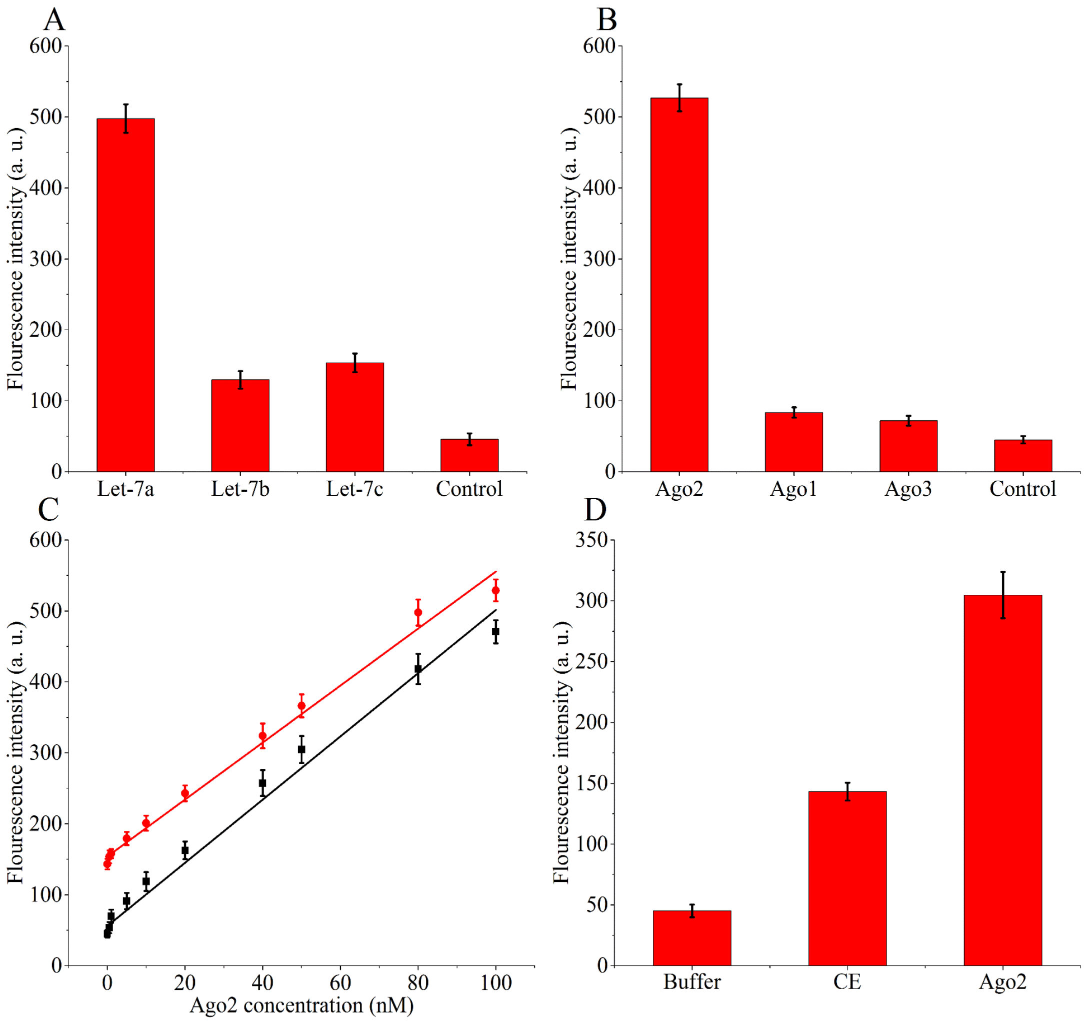

Fig. S4 (A) Fluorescence spectra of the probe after incubation with 150 nM Ago2 and 0.2 nM different siRNA of let-7 family. (B) Comparing the signals from the Ago2 (150 nM) and other analogues of Ago2 (150 nM each) treated with 0.2 nM Let-7a and probe. All the data are taken from

independent experiments with repetition for at least three times, and the presented data are the results of averaging. (C) Relationship between the fluorescence intensity and the concentration of Ago2 in buffer (black point, interaction with 0.2 nM Let-7a) and in cell extract (red point, without additional adding Let-7a). (D) Fluorescence spectra of the probe after incubation with Ago2 in different conditions: buffer, cell extract (CE), and 50 nM Ago2 in buffer. The concentration of Ago2 in CE is calculated according to the fluorescence intensity and the regression equation of the standard curve in the insert of Fig. S3D. The concentration of Ago2 in CE is 19.6 nM. All the data are taken from independent experiments with repetition for at least three times, and the presented data are the results of averaging.

The specificity of the proposed strategy was evaluated with two control experiments that involved (1) three members of the Let-7 family (Let-7a, Let-7b, and Let-7c), with only one or two nucleotide differences out of 22 nucleotides between them; (2) two other analogues of Ago2 (argonaute1 (Ago1), and argonaute3 (Ago3)), respectively. Fig. S4A shows the fluorescence changes for the Let-7a and the other two siRNAs treated with Ago2 and probe. These results clearly demonstrate that the detection approach shows a high selectivity toward the siRNA. This high specificity with the mismatch discrimination ability was derived from the recognition part of Strand2-siRNA hybridization step, which was dominated by the hybridization stability between the siRNA and the recognition part. We next test the selectivity of the Ago2 with other proteins. From Fig. S4B, it was found that Ago2 results in an obvious change in the fluorescence, while there was nearly a negligible fluorescent change in the presence of other analogues. This high specificity with the proteins was derived from the recognition ability of Ago2 to Let-7a.

2.2 Regular monitoring of Ago2/Let-7a complex activity in cell extracts

To test the stability of the probe before the intracellular and cell extract usage, the probe was incubated in PBS by using DNase I as the model for the cleavage assay. Compared with Ago2/Let-7a fluorescence intensity, the mixture of probe and 1 U mL^{-1} of DNase in PBS showed negligible fluorescence after incubation for 120 min (Fig. S1D), indicating excellent protection against nuclease cleavage.

To verify the feasible of the probe used in the assay of Ago2/Let-7a complex in crude cell extracts, we measure the probe's response in buffer, cell extract, and, finally, extract to which an excess of exogenous Ago2 or Let-7a have been added. We find that the probe functions effectively even when employed in media as crude cell extract with a sample volume of 10 μL. Table S2 shows the experimental results obtained in Ago2-spiked cell extracts samples. The results were compared with the standard curve in Fig. S3D insert and the results are given in Fig. S4C. If there is little or even no matrix interference in cell extracts, the curve from the dilution experiment should be coincident with or parallel to the standard curve of Ago2. Due to the fact that the cell extracts contain Ago2, the line of Ago2 in cell extracts is above the line of Ago2 in buffer. Performing a sequential addition and the fluorescence measurement series, measuring buffer, extract, and after further addition of different concentrations of Ago2, we determined the endogenous Ago concentration to be 21.4 ± 1.2 nM (Mean ± SD, Table S2) according to the fluorescence intensity and the regression equation of the standard curve in Fig. S3D insert. While this is in close agreement with the results of direct detection in cell extracts (19.6 nM, CE in Fig. S4D). From Table S2, Ago2 concentration recoveries of 100.6-115.9% were also achieved.

Table S2. Ago2 concentration calculation in CE according to the standard curve and the results in the insert of Fig. S3D.

**Sample**	Ago2		
	Added (nM)	Found (nM)	Recovery (%)
**1**	0	19.6	
**2**	0.5	21.9	109.2
**3**	1	22.9	111.8
**4**	5	27.7	115.9
**5**	10	32.6	115.2
**6**	20	42.0	112.4
**7**	50	69.7	100.6

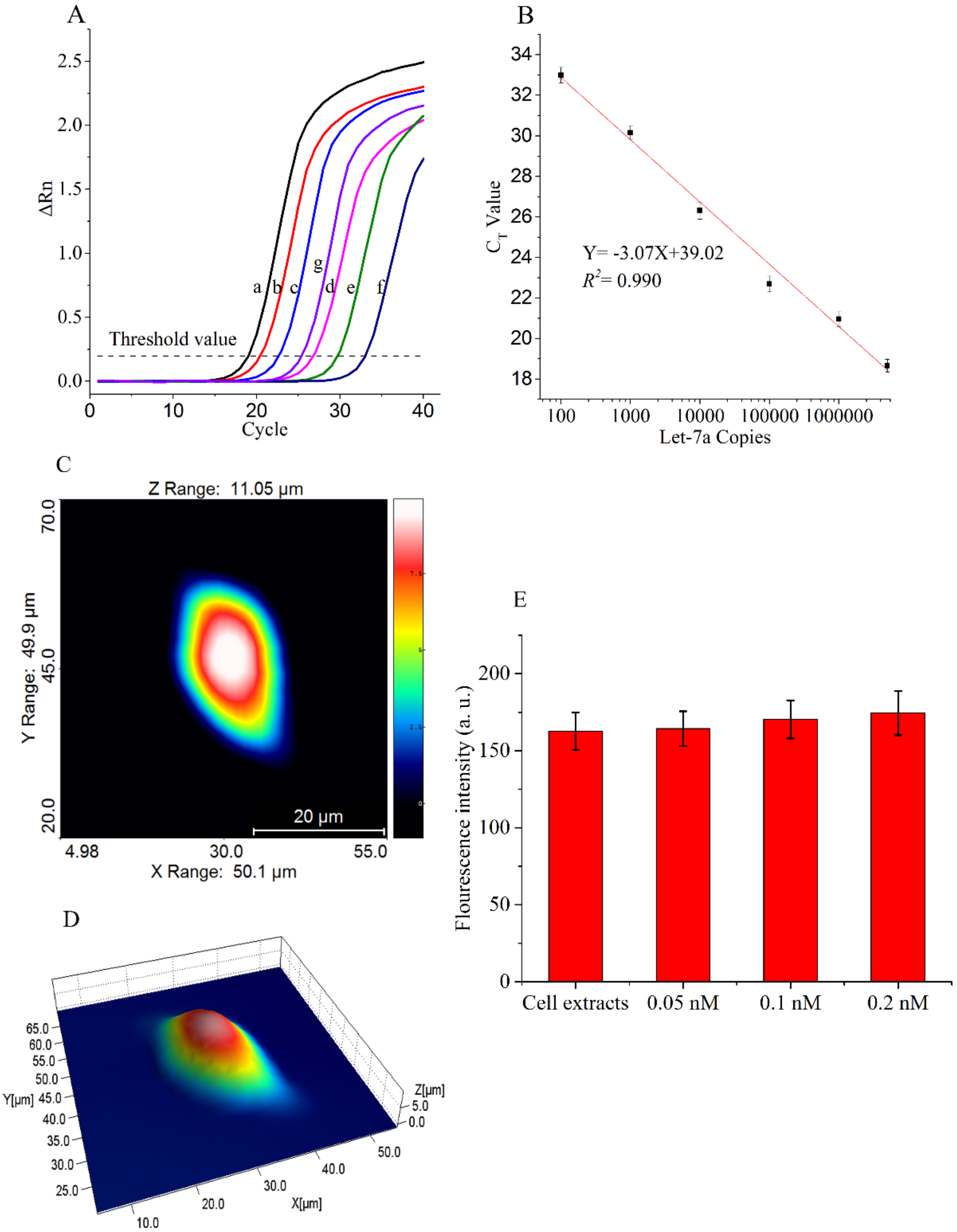

Fig. S5 The calculation of the Let-7a concentration in single-cells and the effect of the adding of different concentration of Let-7a in A549 cell extract. (A) Determination the average number of siRNA inside A549 cells by qRT-PCR. qRT-PCR curves of Let-7a with different copies: (a) 5×10^6; (b) 1×10^6; (c) 1×10^5; (d) 1×10^4; (e) 1×10^3; and (f) 1×10^2, respectively. The threshold value was 0.2. The curve (g) is the copies of the Let-7a in 1000 A549 cell extracts; (B) Plots of C_T values in qRT-PCR assay of Let-7a vs. logarithm of the copies of Let-7a. Error bars were obtained from three parallel experiments; (C) The SICM 2D image of A549 single cell simulated by using the SPIP software; (D) The SICM 3D image of A549 single cell simulated by using the SPIP software; (E) Fluorescence intensity in cell extract with the adding of different concentration of Let-7a (0 nM, 0.05 nM, 0.1 nM and 0.2 nM, respectively).

We next measure the fluorescence intensity in cell extract with the adding of different concentration of Let-7a. We first calculated the Let-7a concentrations in A549 cells as about 0.23 nM by using qRT-PCR method and scanning ion conductance microscopy (SICM) technology (Equation 3 in supporting information and Fig. S5, qRT-PCR for Let-7a amount detection and SICM for A549 volume measure). Fig. S5 shows the experimental results obtained in Let-7a spiked cell extracts samples and the results were compared with the fluorescence intensity obtained in cell extract (column *Cell extracts* in Fig. S5E). In the addition of different concentrations of Let-7a, no obvious changes were displayed. This phenomenon may contribute to the adequate concentration of Let-7a in the cell extracts. Since 0.2 nM Let-7a can give the maximum responses in the absence of 1 to 150 nM Ago2 (Fig. 1F-orthogonal experiments) and the cell extract contains Let-7a with a concentration of 0.23 nM, the additional added Let-7a may not give higher fluorescence intensities.

3. ADDITIONAL FIGURES

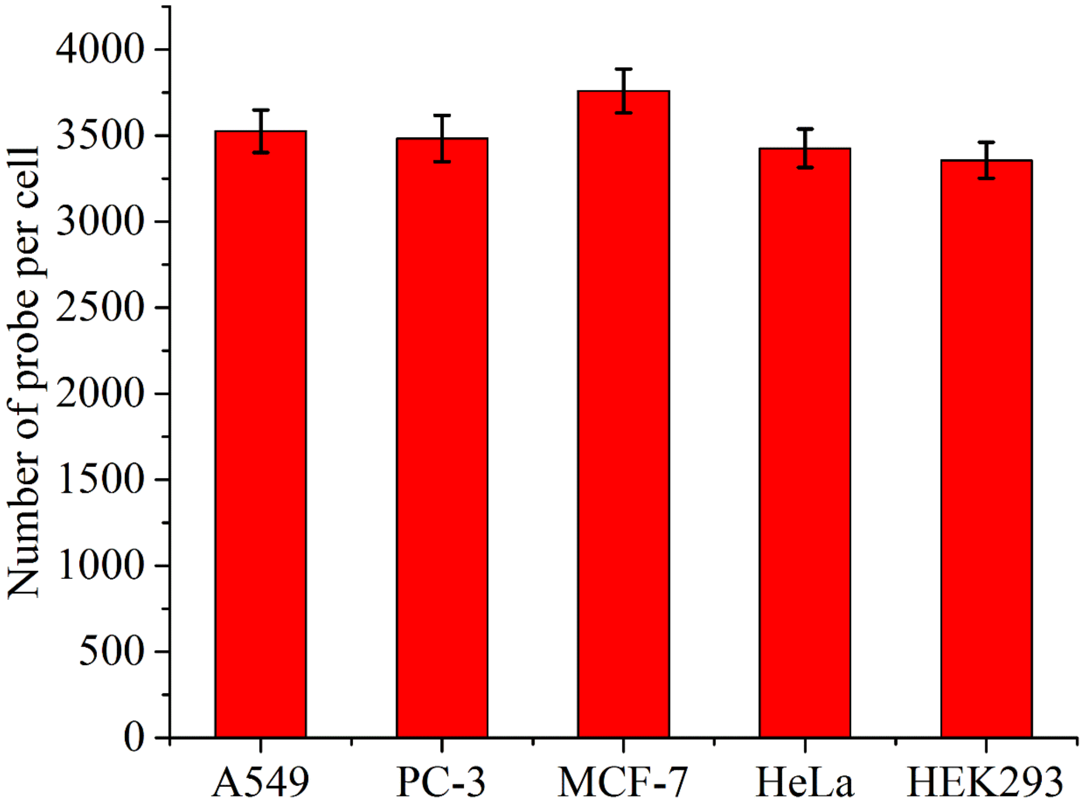

Fig. S6 The uptake amounts of probe in different types of cells.

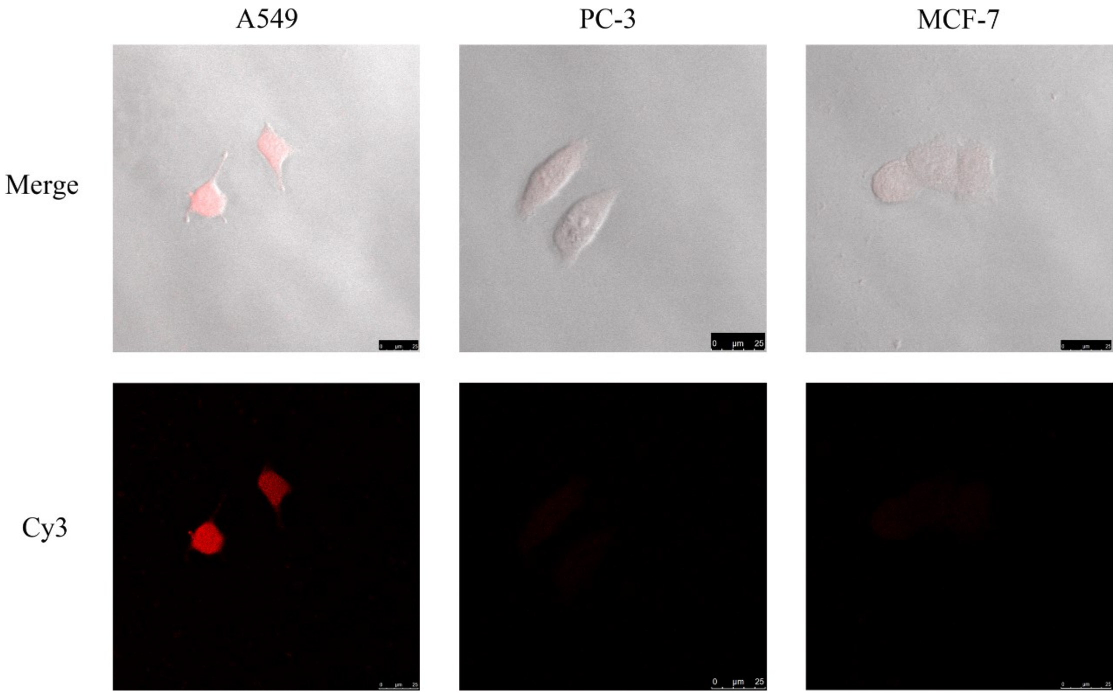

Fig. S7 Confocal images of A549, PC-3, and MCF-7 cells after incubation with 25 μL of probe for 90 min (scale bar 25 μm).

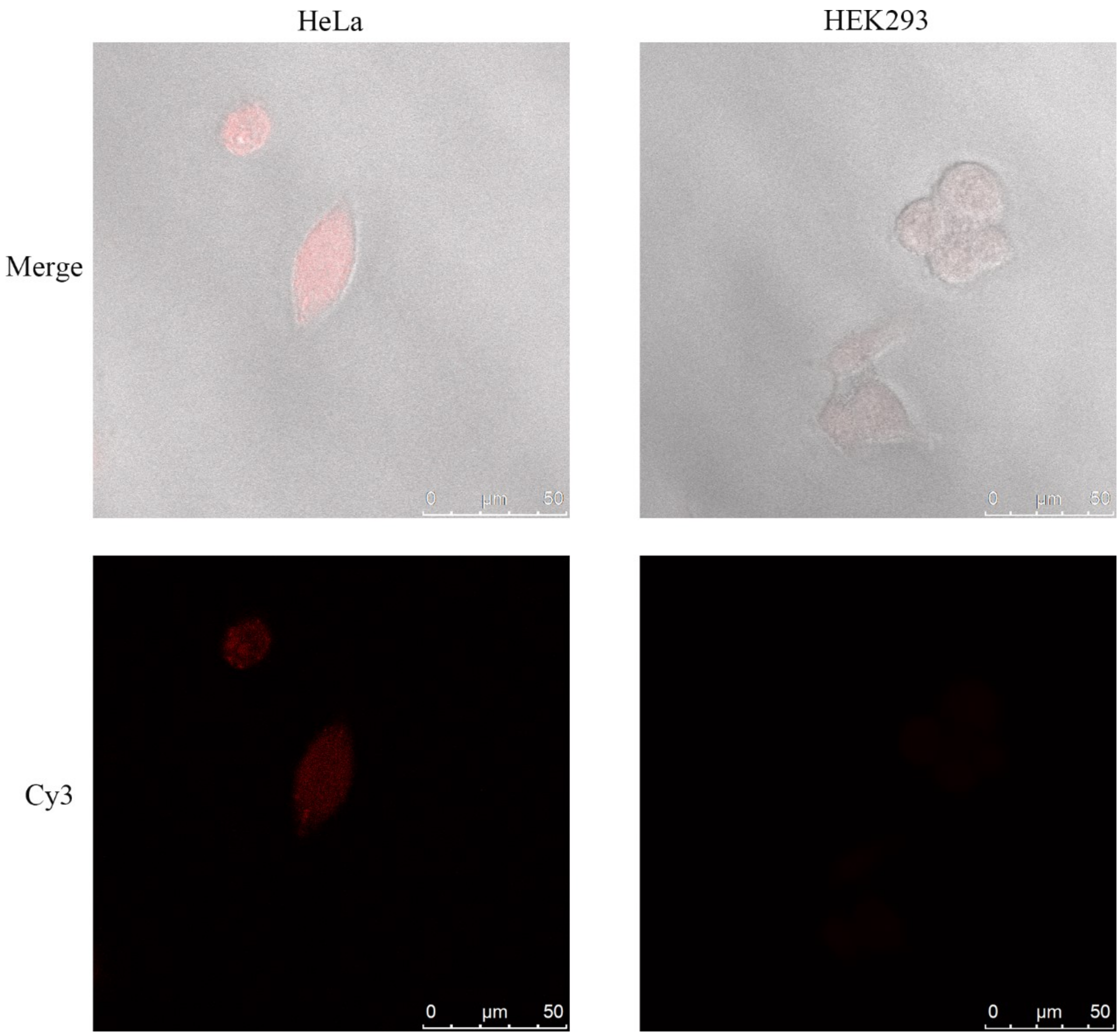

Fig. S8 Confocal images of HeLa, and HEK293 cells after incubation with 25 μL of Ago2/miR-21 targeted probe for 90 min (scale bar 50 μm).

4. REFERENCES

[1] W. Haiss, N. T. K. Thanh, J. Aveyard, D. G. Fernig, *Anal. Chem.* **2007**, *79*, 4215-4221.

[2] a) R. Qian, L. Ding, L. Yan, M. Lin, H. Ju, *J. Am. Chem. Soc.* **2014**, *136*, 8205-8208; b) K. C. Grabar, R. G. Freeman, M. B. Hommer, M. J. Natan, *Anal. Chem.* **1995**, *67*, 735-743.

[3] a) Y. Li, C.-y. Zhang, *Anal. Chem.* **2012**, *84*, 5097-5102; b) K. Ui-Tei, Y. Naito, S. Zenno, K. Nishi, K. Yamato, F. Takahashi, A. Juni, K. Saigo, *Nucleic Acids Res.* **2008**, *36*, 2136-2151; c) C. I. Chang, J. W. Yoo, S. W. Hong, S. E. Lee, H. S. Kang, X. G. Sun, H. A. Rogoff, C. Ban, S. Kim, C. J. Li, D. K. Lee, *Mol. Ther.* **2009**, *17*, 725-732.

[4] S. Baluchamy, N. Sankar, A. Navaraj, E. Moran, B. Thimmapaya, *Oncogene* **2006**, *26*, 781-787.
